# Geographic assessment of access to health care in patients with cardiovascular disease in South Africa

**DOI:** 10.1186/s12913-018-3006-0

**Published:** 2018-03-22

**Authors:** Thandi Kapwata, Samuel Manda

**Affiliations:** 1 Environment and Health Research Unit, South African Medical Research Council, Johannesburg, South Africa; 2 Biostatistics Research Unit, South African Medical Research Council, Pretoria, South Africa; 3 Division of Epidemiology and Biostatistics, School of Public Health, University of Witwatersrand, Johannesburg, South Africa

**Keywords:** Cardiovascular disease, Geographic information systems, Healthcare access

## Abstract

**Background:**

Noncommunicable diseases (NCDs) including cardiovascular diseases (CVDs), diabetes, cancer and chronic lung disease are increasingly emerging as major contributors to morbidity and mortality in developing countries. For example, in South Africa, 195 people died per day between 1997 and 2004 from CVDs related causes. Access to efficient and effective health facility and care is an important contributing factor to overall population health and addressing prognosis, care and management CVD disease burden. This study aimed to spatially evaluate geographic health care access of people diagnosed with CVD to health facilities and to evaluate the density of the existing health facility network in South Africa.

**Methods:**

Data was obtained from the National Income Dynamics Study (NIDS) conducted in 4 waves (phases) between 2008 and 2014. The participants who responded as having heart problems that were diagnosed by a health practitioner were extracted for use in this study. Network analyst in ArcGIS ® was used to generate a least-cost path, which refers to the best path that one can travel. The residential locations of participants diagnosed with heart problems were put into the network analysis model as origins and the location of health facilities were destinations. District averages were used to protect the identity of studied participants.

**Results:**

There were a total of 51, 42, 43, 43 health districts out the 52 that had recorded subjects with a heart condition in the 2008, 2010–2011, 2012 and 2014–2015 waves, respectively. The mean distance from a case household to a health facility per wave was 2, 2.3, 2.1 and 2.1 km in 2008, 2010–2011 and 2014–2015 respectively. The maximum individual distances travelled per wave were 41.4 km, 40,5 km, 44,2 km and 39.6 km for the 2008, 2010–2011, 2012 and 2014–2015 waves respectively. For district level analysis, participants with CVD residing in the districts found to be among the poorest in the country travelled the longest distances. These were located in the provinces of Limpopo and KwaZulu Natal. It was also found that districts with large proportions of their population living in rural settings had among the lowest densities of health facilities. Significant percentages of study participants were exposed to numerous CVD risk factors, the commonly reported one being high blood pressure. A lack of regular exercise was also commonly reported in each of the waves.

**Conclusion:**

A lack of accessible healthcare in already impoverished municipalities could result in an increase lack of timely diagnosis, CVD case management. This could result in increased CVD-related morbidity and mortality. GIS methods have the potential to assist national health programs to develop policies that target issues such as areas or populations being underserved by health facilities and populations that must travel long distances to receive healthcare. These policies will be key in preventing and controlling the emerging CVD burden through an accessible primary healthcare system for early detection and case management.

**Electronic supplementary material:**

The online version of this article (10.1186/s12913-018-3006-0) contains supplementary material, which is available to authorized users.

## Background

Noncommunicable diseases (NCDs) are major contributors to morbidity and mortality worldwide [1]. For instance, an estimated 17.5 million people died from cardiovascular diseases (CVDs), accounting for 31% of global deaths, in 2015 [1]. In particular, cardiovascular diseases are responsible for 10% of disability adjusted life years (DALYs) lost in low- and middle-income countries (LMICs) and with 82% of CVD deaths occurring in less economically developed countries [2, 3]. In developing countries, emergence of the NCDs are adding huge burden on health care systems and resources where there is already appalling burden of infectious (TB and HIV), and perinatal maternal nutritional-related diseases [4, 5]. The rise in CVD related mortality in LMICs has been attributed to upward trends in the prevalence of CVD risk factors and greater exposure to risk factors [3]. Adults of working age are most affected by CVD and therefore CVD has a huge economic impact on individuals, households and countries [6]. A number of these risk factors including high blood cholesterol, high blood pressure, smoking, lack of physical activity, an unhealthy diet and stress can be controlled [7].

South Africa, with 195 CVD-related deaths per day between 1997 and 2004, has similarly been battling the emergence of NCDs epidemic; heart disease and stroke which are now becoming the leading causes of mortality after HIV ([8]. In recently released mortality statistics, ischemic heart and cerebrovascular disease accounted for 7.6% of all causes of death [9]. The country’s National Department of Health released a strategic plan outline aimed at reducing NCD morbidity, mortality and associated risk factors prioritizes cardiovascular diseases (CVDs) [10]. The implementation of the National NCD strategies and plans could be supported by a number of research studies including robust epidemiological assessments of populations and places at high risk of exposure to adverse CVD outcomes and risk factors and assessment of available data sources. Early identification of CVD conditions, management and control of CVD patients is a major public health concern. Outcomes of CVD are mostly dependent on access to efficient and effective primary health care. This includes access to health practitioners, regular medical screening, blood pressure lowering drugs and cholesterol lowering drugs [11]. A well-functioning and accessible primary healthcare system is key to achieving a reduction in CVD burden and contributing to overall population health [3, 12]. Communities being able to access to health care can prevent or reduce expensive specialty care, delayed presentation or even death as a result of CVD or CVD risk factors [13, 14].

Geographical information systems (GIS) software is a tool that has previously been used to model accessibility to healthcare services [15–18]. Access to healthcare services is influenced by several factors. These include 1) geographical accessibility – the distance or time between a health care and a user; 2) availability – having access to appropriate health care providers with the necessary materials and equipment; 3) financial accessibility – pricing of health services, ability of users to pay for services, transport costs to users and 4) acceptability – receptiveness of health service providers to the social and cultural practices of the communities in which they serve [19]. This study focuses on geographical accessibility because studies in developing countries have shown that physical proximity of health services is strongly associated with utilization of health services [20–22]. Past research has also found significant associations between heart disease and geographic access to hospitals [23, 24]. This type of research is of particular interest to health systems policy makers and public health planners because geographic access (distances, travel times and catchments) informs spatial planning and health policy making [25].

Therefore, the aim of the study was to spatially evaluate geographic health care access of people diagnosed with CVD in South Africa to health facilities and to evaluate the density of the facility network. The spatial location of healthcare facilities and residences of participants diagnosed with CVD were incorporated into a GIS to allow for the geographical defining of accessibility based on travel times and distances. This GIS framework will enable the quantifying of population access to health resources at a district level.

## Methods

Health status data obtained from The National Income Dynamics Study (NIDS) was used to extract participants diagnosed with CVD. GIS analysis was used to incorporate the spatial location of these participants and the location of health facilities to estimate the distance travelled to the nearest health facility along the road network. Network distance to the nearest health facility is a better estimator of distance than the Euclidean distance because it takes the actual road network into account. Further analysis was performed to determine the distribution of health facilities within the various districts.

### NIDS data

The National Income Dynamics Study (NIDS) was initiated in 2008 by the South African Presidency. It was an intensive effort to track changes in the well-being of South Africans by closely following about 28,000 people over a period of years. The NIDS survey is the first national panel study to document the dynamic structure of a sample of household members in South Africa and changes in their incomes, expenditures, assets, access to services, education, health, and other dimensions of well-being. A total of 7305 households were enrolled in the study and participants are tracked as they move to different locations. This ensures that the movement of household members will be accurately captured in subsequent waves of the study.

A stratified, two-stage cluster sample design was employed in sampling the households to be included in the base wave [26]. The original NIDS sample was obtained in 2008 and 10,367 dwellings were selected from 400 Primary Sampling Units across the country [27]. Of those dwellings, 7296 were found to be eligible and successfully interviewed [27]. Within the successfully interviewed households, 31,144 individuals were identified. Non-resident members of households were excluded from the sample, a resident member was defined as an individual that usually resided at the dwelling four nights a week. A final count of 28,226 individuals comprised the continuing sampling members [27]. Since the beginning of the survey, there have been no refreshment samples added.

The design of the NIDS study envisaged data collection every 2 years. Therefore, the first wave of NIDS was conducted in 2008, the second from 2010 to 2011, the third in 2012 and the last one between 2014 and 2015 [28–31]. The questionnaire completed by adults recorded information about demographics, education, employment, income, health, well-being, numeracy, anthropometric data. One of the health questions that was captured asked participants whether they have ever been told by a doctor, nurse or health care professional that they have heart problems. Since CVD is a series of heart diseases, the participants who responded with yes were extracted for each wave to use in this study.

### Roads data

The national road network data was obtained from the National Geo-spatial Information department, a national South African mapping organization [32]. Each road segment was assigned a class (regional primary, district or local distributor). The speed limit assigned to each road segment was the maximum legal limit for the class attributed to it.

### Health facilities

GPS coordinates of health facilities in South Africa were obtained from the District Health Information System (DHIS) that was adopted by the South African National Department of Health in the year 2000. Data regarding the location of public health facilities (gps co-ordinates, physical address and postal address), type of facility (for example clinic, hospital and mobile structure) and the services provided are captured, validated and updated monthly. This ensures the correctness, completeness, and consistency of the national health facilities data repository.

### Network analysis

#### Origin-destination (OD) cost matrix

A network is a set of interconnected lines that can represent features such as rivers, roads, waterlines, telecommunication lines etc. In ArcGIS, transportation networks can be modelled by using a network dataset comprising of a road network. This type of analysis models movement of people or goods that are transported along the road network. The OD cost matrix solver, an application in ArcGIS ® [33], finds and measures the least-cost paths along the road network from multiple origins to multiple destinations. This was used to generate a least-cost path, which refers to the best path that one can travel. The output is a matrix of the shortest travel times and distances to all destinations from all origins. The residential locations of participants diagnosed with heart problems were put into the network analysis model as origins and the location of health facilities were destinations.

The OD cost matrix solver was based on a few assumptions. The first is that participants will choose the shortest path to the nearest health facility [34]. The second is that the shortest travel time to a health facility is the shortest path to health care [34]. Third, the network analysis model assumed that survey participants experienced the same travel conditions therefore variation in factors that influence travel time such as day of the week, time of day, traffic patterns and weather are held constant [35]. Lastly, although a hierarchal speed limit classification system was used in the analysis, travel distances could not account for the variability in road surface types which could affect speed [35].

#### Point density analysis

A point density analysis was performed using ArcGIS ®to assess the spatial patterns of hospitals in all the provinces. This tool calculates a magnitude-per-unit area from point features that fall within a specified boundary. Once the boundary is defined, the number of points that fall within is totaled and divided by the area of the neighborhood. The output of this analysis is a density surface that shows the distribution of health facilities across the various districts. It is therefore possible to evaluate the availability of health facilities by identifying districts with a higher numbers of health facilities and those with a poor distribution of facilities.

## Results

A large number of study participants who were diagnosed with heart problems were from the African population group and mostly female (Table 1). The highest percentages of participants were in the age groups 45–60 years old and older than 60 years old. Several respondents were diagnosed with risk factors, high blood pressure being the most prevalent. A lack of regular exercise was also commonly reported in each of the waves.

**Table 1:** Baseline characteristics of participants in each of the four waves

		2008 Wave ($\frac{n\ }{N\ }$)	2010–2011 Wave ($\frac{n\ }{N\ }$)	2012 Wave ($\frac{n\ }{N\ }$)	2014–2015 Wave ($\frac{n\ }{N\ }$)
Age group	15–18	9 1869	- 1814	9 2404	13 2669
	19–45	132 8302	69 9862	116 10,152	103 10.237
	45–60	186 3298	124 3588	191 3739	136 3630
	> 60	163 3400	133 6614	185 6166	138 5671
Gender	Female	350 9342	253 10,316	364 11,128	300 13,291
	Male	140 6288	73 7304	137 7569	90 9461
Population group	African	326 12,246	219 14,626	343 15,387	297 18,889
	Asian/Indian	18 223	16 193	19 193	9 209
	Mixed race	90 2216	59 2248	101 2585	63 3134
	White	53 909	32 549	38 532	21 519
Respondent was diagnosed with high blood pressure?	No	217 13,073	140 15,374	193 15.430	158 18,193
	Yes	272 2490	185 2141	308 3222	129 2165
Respondent was diagnosed with diabetes?	No	414 14,920	250 16,875	394 17,766	321 21,433
	Yes	70 607	76 654	105 858	50 601
Respondent was diagnosed with a stroke?	No	462 15,371	299 17,413	460 18,463	349 22,355
	Yes	24 156	25 144	41 205	34 220
How regularly does the respondent exercise?	Less than once a week	35 903	12 1313	44 1304	16 1606
	Never	369 10,871	227 12,319	362 13,399	310 15,730
	Once a week	18 867	12 894	28 770	10 1064
	Three or more times a week	50 1926	20 1409	47 2262	37 3036
	Twice a week	18 947	24 832	20 948	16 1283
Does the respondent smoke?	No	382 12,277	253 14,181	395 15,441	315 18,460
	Yes	106 3279	44 2652	106 3239	75 4267
BMI of respondent^a^	Underweight	28	68	133	112
	Normal	136	180	228	156
	Overweight	90	63	117	78
	Obese	168	15	23	30

Notes: ^a^BMI calculated from average of 3 weight and 3 height measurements taken for consenting participants, individuals with missing data for any of the above questions not included in table, n = participants diagnosed with CVD, N = total number of participant enrolled in study

The wave conducted in 2014–2015 had the largest sample size of participants enrolled in the study. The furthest mean distance from participant residence to the nearest health facility was in the 2010–2001 wave (2.34 km) and maximum individual distance travelled in all waves was 44.20 km in Mopani Limpopo (Table 2). The largest number of participants with CVD was recorded in the 2012 wave (477).

**Table 2:** Mean travel distance to health facility by wave

Wave	Total participants in study	Total participants with CVD	Mean (km)	Std dev	Min (km)	Max (km)	District of Residence of participant with maximum distance	95% Confidence Interval
2008	16,871	470	2.0	4.3	1.6E-03	41.4	Frances Baard (Northern Cape)	1.52–2.39
2010–2011	21,880	305	2.3	5.2	2.0E-03	40.5	Fezile Dabi (Free State)	1.65–3.05
2012	22,466	477	2.1	4.9	1.1E-03	44.2	Mopani (Limpopo)	1.57–2.56
2014–2015	26,819	375	2.1	4.9	1.9E-03	39.6	Thabo Mofutsanyane (Limpopo)	1.55–2.67

**Fig. 1:**
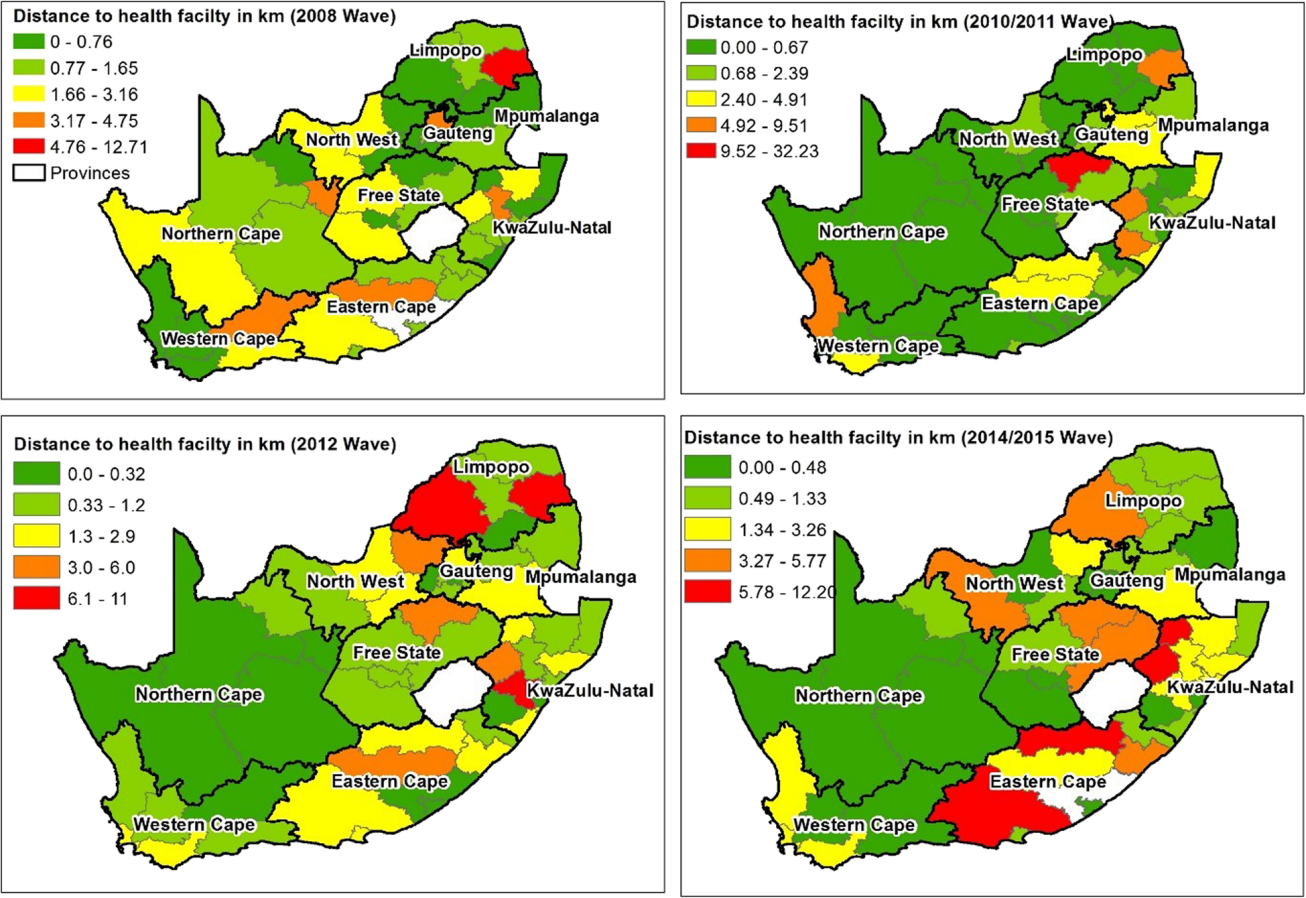
Average distances to health facility by district

**Fig. 2:**
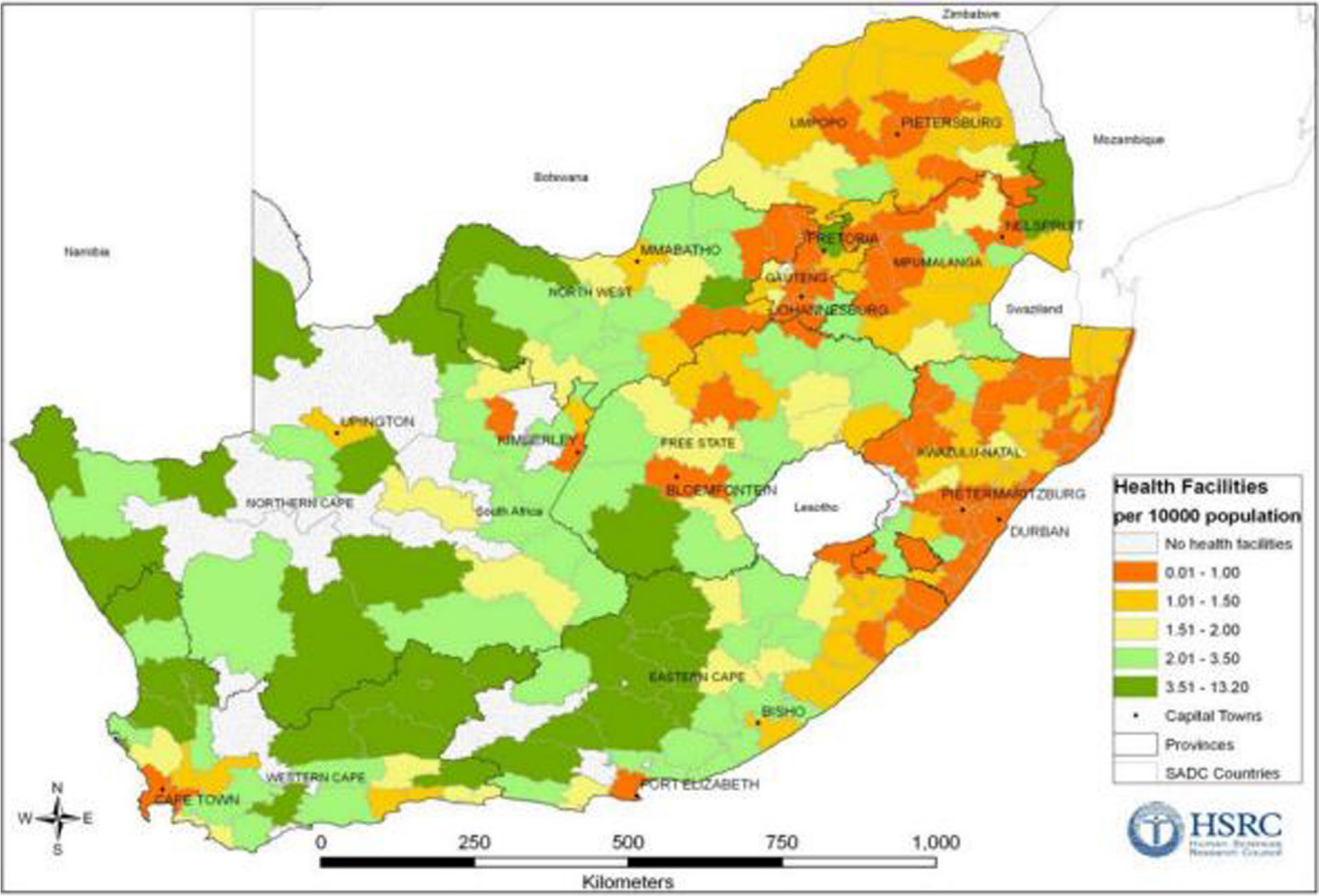
Number of health facilities per 100,000 population (Source: Human Sciences Research Council, 2012)

The mean distance that study participants would have had to travel from their residence along the road network to the nearest health facility are shown in Fig. 1 at the district level. For the 2008 and 2012 waves, heart patients in Mopani and Waterberg in Limpopo had the longest mean travel times to a health facility at 12.7 and 11.4 km respectively. For the waves conducted in 2010–2011, participants in Fezile Dabi district municipality in the Free State province had the longest mean travel distance (32.2 km), which was also the longest travel time across all the waves. For the 2014–2015 wave, participants with CVD in Uthukela district in KwaZulu Natal province had the longest mean travel distances (12.20 km). Additional file 1 presents four graphs that show mean distance in kilometers to a health facility from a household with subject diagnosed with a heart condition for each of the waves by district. An estimation of the density of health facilities per 10,000 population in South Africa presented at municipality level in Fig. 2 shows that KwaZulu Natal province had the lowest number of health facilities per 10,000 population. The majority of the province’s municipalities have 0.01–1.50 health facilities per 10,000 population. Figure 2 also shows that Limpopo province had the second lowest coverage of health facilities per 10,000 people, more than 70% of the province’s local municipalities had less than 1.50 health facilities per 10,000 people [36].

**Fig. 3:**
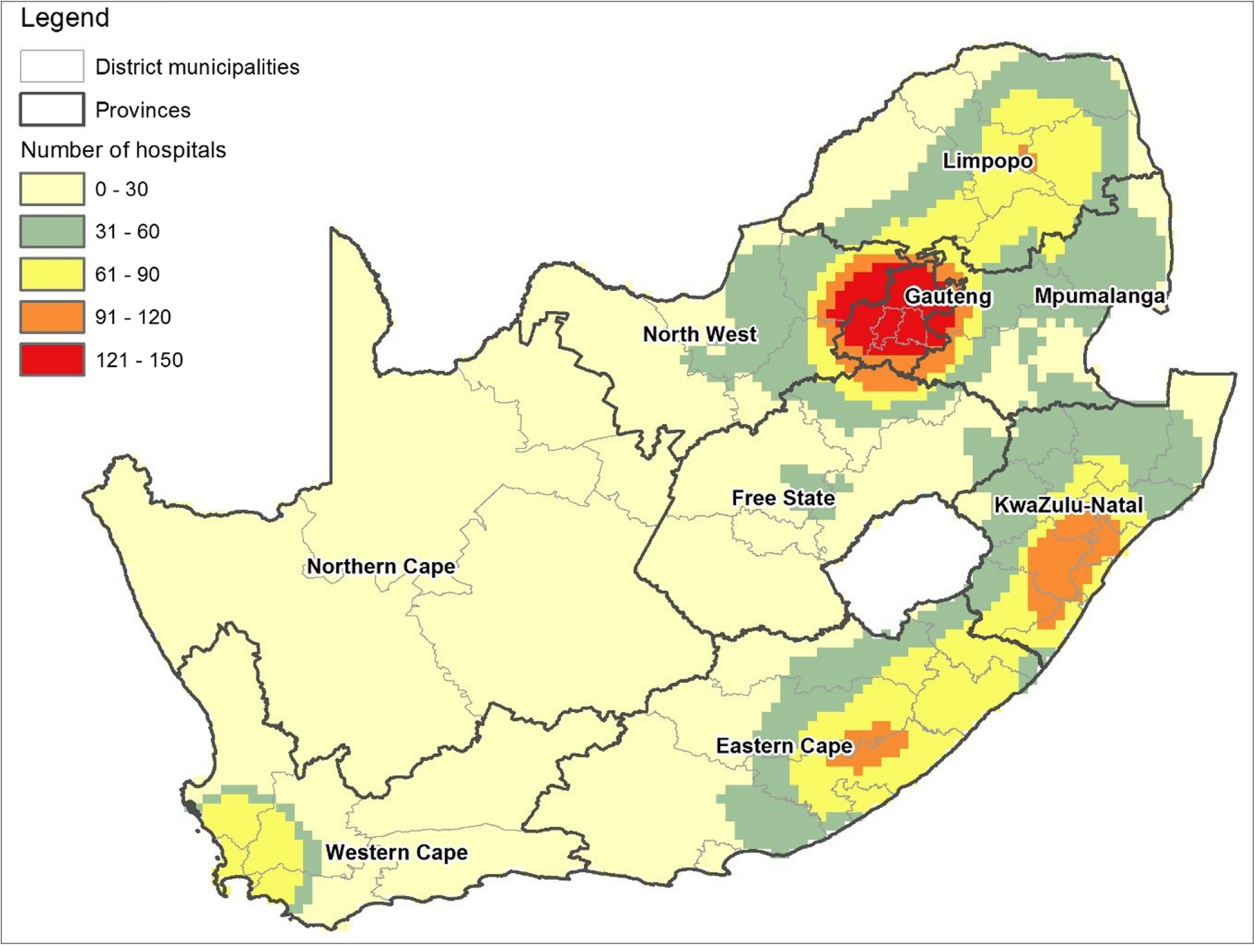
Density of health facilities in municipal districts of South Africa

A point density analysis of the health facilities nationally shows that the distribution is not even in all the provinces (Fig. 3). The Northern Cape had the lowest density of health facilities, followed by the Free State. It was established in earlier analysis that the longest individual distances travelled to health facilities were by participants residing in these provinces (2008 and 2010–2011 waves). Although Gauteng is the smallest province, it has the highest density of hospitals and is therefore the province best covered by the health network.

## Discussion

This study used data from four waves of the NIDS to evaluate health care access by analyzing distance travelled by participants diagnosed with heart disease from their residences to the nearest public health facility. We also assessed the distribution of health facilities nationally. The results from this study could provide useful information that can be used in health service delivery planning and the assessment of access to healthcare.

Results showed that significant percentages of participants were exposed to CVD risk factors. Observational studies involving 1 million individuals found that for every 20 mmHg systolic or 10 mmHg diastolic increase in BP, mortality from both heart disease and stroke doubled [37, 38]. High blood pressure (HBP) was the most commonly reported risk factor in all four waves (55.62%, 56.92, 61.48% and 44.95%). Therefore, these individuals are at a high risk of succumbing to heart diseases or strokes. This high prevalence of HBP in people who have heart disease supports the global statistics released by WHO [39] that increased bold pressure is the leading risk factor for heart disease worldwide. In South Africa, the risk of death from HBP has increased by 25% in less than a decade [40].

Regular blood pressure screening and treatment are important aspects in the control of HBP however this can be hampered by poor access to health facilities leading to poor outcomes in patients. Individuals with HBP require consistent evaluation by medical practitioners including physical examinations and routine laboratory tests in order to monitor the effectives of treatment or lack thereof, to assess presence of organ damage and to monitor comorbid conditions [41]. Once blood pressure is at an optimum level, follow up visits can be at 3 or 6-month intervals [41]. A study by [42] reported a positive association between a shorter distance of travel to a health facility and adherence to hypertensive medication. Therefore, participants in the NIDS survey are likely to increase time intervals between health facility visits or default on HBP treatment if they must travel long distances to health care. Another study that demonstrates a link between distance to healthcare and HBP was done by [43]. They found that travelling long distances to prenatal care was associated with increased risk of having HBP during pregnancy.

Lack of physical activity is one of the lifestyle risk factors of heart disease and the majority of respondents in all waves reported that they never exercise (75.31%, 76.95, 72.26% and 79.69%). Studies have found that regular exercise can reduce the progression of heart disease and substantially lowers the risk of recurrent cardiovascular events [44, 45].

A systematic review found that 71% of all studies reported that a high volume of hospitals is associated with better outcomes across a wide range of conditions [46]. The results of our analysis show that high volumes of health facilities are concentrated in provinces with high economic activities (Gauteng), disadvantaging poor communities. A high number of districts are underserved by health facilities due to the low density of facilities (less than 30 facilities per district). This emphasizes poor health network coverage experienced by study participants with CVD living in those districts, delayed access to medical care in patients with heart disease increases myocardial damage and mortality [14]. Those who are reliant on public transport are particularly affected and this is the most common form of transport in South Africa [47]. Health facilities are concentrated in provinces with high economic activities (Gauteng), further disadvantaging poor communities. The concentration of public health facilities in urban areas shows that they are prioritized by health systems development over rural areas.

The performance of an efficient health system is commonly gauged by equity in health service provision [20]. Equity can be measured by the distribution, access and utilization of health services [20]. This study shows that the distribution of health facilities is not uniform and therefore equity with regards to access is compromised. The Northern Cape and Free State provinces had among the lowest density of health facilities and both had longest individual travel distances for 2 waves (41.4 and 40.5 km, for 2008 and 2010–2011 waves respectively). Limpopo had the longest individual travel distance for 2 waves (44.2 and 39.6 km for 2012 and 2014–2015 waves respectively). A study conducted by [48] in KwaZulu Natal province involving 404 participants reported that patients with XDR TB in three districts travelled between > 10 – 50 km to the health facility at which they were diagnosed. Our results show that the individual longest distance to a health care are within this range.

These provinces also had the highest unemployment rates amongst adults aged 35–64 years in 2015 Statistics South Africa [49]. This is of concern because studies have shown that individuals of lower socioeconomic status are more vulnerable to heart disease due to increased exposure to risk factors [6, 50–53].

Results showed disparities in access to health facilities between the various districts across South Africa. District level analysis showed that Fezile Dabi in the Free State province had the furthest mean travel distance to the nearest health facility (32.23 km for the 2010–2011 wave). Mopani and Waterberg (Limpopo) and Uthukela (KwaZulu Natal) districts also had the highest mean travel times for the 2008, 2012 and 2014–2015 waves (12.7,11.38 and 12.20 km respectively). These districts have large proportions of their populations living in rural areas and rural communities are usually among the poorest in the country. This study provides evidence that rural communities are at a disadvantage due to travelling long distances to seek medical care.

The main limitation of our study was that although the GPS co-ordinates of residential location for all survey participants were available, a data confidentiality agreement limited us to district level reporting of NIDS survey data and subsequent analysis.

## Conclusion

This study analyzed the distance of public health facilities from the residential locations of participants diagnosed with CVD. Results showed that several districts were found to have low densities of public health facilities. A lack of accessible healthcare in already impoverished municipalities could result in an increased lack of timely diagnosis and poor CVD case management. Analysis further showed that the poor suffering from CVD tend to reside furthest from the nearest health facility resulting in unreasonable travel burdens. The inability to bear travel costs therefore constrains them in terms of accessing health care. Therefore, it is evident that proximity to a health facility and financial assistance are important factors in assisting the poorest populations. Spatial analysis using GIS can be used for decision making by South Africa’s national health systems programs to develop policies that address issues such as the poor distribution of health facilities, particularly in rural areas, that results in areas or populations being underserved by health facilities. GIS can also be used for the identification of populations that must travel long distances to receive healthcare. This is crucial in policy development regarding equity of access to health care. The findings of this study provide evidence to health systems decision makers that future resource planning should include increasing the number of health facilities to improve accessibility. Furthermore, these facilities should ideally be located in underserved districts. These policies will be key in preventing and controlling the emerging CVD burden through an accessible primary healthcare system for early detection and case management.

## Additional files

Additional file 1: Graphs showing mean distance to a health facility from a household with subject diagnosed with a heart condition for each of the waves by district. Part 1: Codes of district municipalities. Map of South Africa displaying district codes. Part 2: Mean distance in kilometres to a health facility from a household with subject diagnosed with a heart condition in the 2008 and 2010–2011 NIDS Survey wave by district, South Africa. Mean distance to health facility by district for each wave. Part 3: Mean distance in kilometres to a health facility from a household with subject diagnosed with a heart condition in the 2012 and 2014–2015 NIDS Survey wave by district, South Africa. Mean distance to health facility by district for each wave. (ZIP 707 kb)

## Abbreviations

CVDs: Cardiovascular diseases
DALYs: disability adjusted life years
DHIS: District Health Information System
GIS: Geographical information systems
HBP: High blood pressure
LMICs: in low- and middle-income countries
NCDs: Noncommunicable diseases
NIDS: National Income Dynamics Study
OD: Origin-destination

## References

[1] World Health Organization Cardiovascular diseases Fact Sheet Available at http://www.whoint/mediacentre/factsheets/fs317/en/. Accessed 29 Mar 2107.

[2] World Health Organization Global Health Risks Report Part 2. Available at http://www.who.int/healthinfo/global_burden_disease/GlobalHealthRisks_report_part2.pdf. Accessed 28 Nov 2017.

[3] Joshi R, Jan S, Wu Y, MacMahon S (2008). Global inequalities in access to cardiovascular health care: our greatest challenge. J Am Coll Cardiol.

[4] Daar AS, Singer PA, Leah Persad D, Pramming SK, Matthews DR, Beaglehole R, Bernstein A, Borysiewicz LK, Colagiuri S, Ganguly N (2007). Grand challenges in chronic non-communicable diseases. Nature.

[5] Mayosi BM, Flisher AJ, Lalloo UG, Sitas F, Tollman SM, Bradshaw D. The burden of non-communicable diseases in South Africa. Lancet. 374(9693):934–47. 10.1016/S0140-6736(09)61087-4 19709736

[6] Reddy KS (2004). Cardiovascular disease in non-western countries. N Engl J Med.

[7] Association AD (2015). Cardiovascular disease and risk management. Diabetes Care.

[8] Heart and Stroke Foundation of South Africa and South African Medical Reseach Council: Heart Disease in South Africa Available at http://www.heartfoundation.co.za/wp-content/uploads/2017/10/CVD-Stats-Reference-Document-2016-FOR-MEDIA-1.pdf. Accessed 29 Mar 2017.

[9] Statistics South Africa, National Census 2011**.** Available at http://www.statssa.gov.za/. Accessed 22 Sept 2017.

[10] South African National Department of Health Strategic Plan**:** 2014**/**15–2018/19**.** Available at https://www.health-e.org.za/wp-content/uploads/2014/08/SA-DoH-Strategic-Plan-2014-to-2019.pdf. Accessed 28 Nov 2017.

[11] Lim SS, Gaziano TA, Gakidou E, Reddy KS, Farzadfar F, Lozano R, Rodgers A (2007). Prevention of cardiovascular disease in high-risk individuals in low-income and middle-income countries: health effects and costs. Lancet.

[12] Guagliardo MF (2004). Spatial accessibility of primary care: concepts, methods and challenges. Int J Health Geogr.

[13] Luo W, Wang F. Spatial accessibility to primary care and physician shortage area designation: a case study in Illinois with GIS approaches. Geographic information systems and health applications. 2003:260–78.

[14] Luepker RV, Raczynski JM, Osganian S, Goldberg RJ, Finnegan Jr JR, Hedges JR, Goff Jr DC, Eisenberg MS, Zapka JG, Feldman HA (2000). Effect of a community intervention on patient delay and emergency medical service use in acute coronary heart disease: the rapid early action for coronary treatment (REACT) trial. JAMA.

[15] Coffee N, Turner D, Clark RA, Eckert K, Coombe D, Hugo G, van Gaans D, Wilkinson D, Stewart S, Tonkin AA (2012). Measuring national accessibility to cardiac services using geographic information systems. Appl Geogr.

[16] Cinnamon J, Schuurman N, Crooks VA (2008). A method to determine spatial access to specialized palliative care services using GIS. BMC Health Serv Res.

[17] Nykiforuk CI, Flaman LM (2011). Geographic information systems (GIS) for health promotion and public health: a review. Health Promot Pract.

[18] Okwaraji YB, Cousens S, Berhane Y, Mulholland K, Edmond K (2012). Effect of geographical access to health facilities on child mortality in rural Ethiopia: a community based cross sectional study. PLoS One.

[19] Munoz UH, Källestål C (2012). Geographical accessibility and spatial coverage modeling of the primary health care network in the Western Province of Rwanda. Int J Health Geogr.

[20] Noor A, Zurovac D, Hay S, Ochola S, Snow R (2003). Defining equity in physical access to clinical services using geographical information systems as part of malaria planning and monitoring in Kenya. Tropical Med Int Health.

[21] Feikin DR, Nguyen LM, Adazu K, Ombok M, Audi A, Slutsker L, Lindblade KA (2009). The impact of distance of residence from a peripheral health facility on pediatric health utilisation in rural western Kenya. Tropical Med Int Health.

[22] Müller I, Smith T, Mellor S, Rare L, Genton B (1998). The effect of distance from home on attendance at a small rural health Centre in Papua New Guinea. Int J Epidemiol.

[23] Yamashita T, Kunkel SR (2010). The association between heart disease mortality and geographic access to hospitals: county level comparisons in Ohio, USA. Soc Sci Med.

[24] Acton QA: Cardiovascular diseases: advances in research and treatment**:** 2011 Edition: ScholarlyEditions; 2012.

[25] Comber AJ, Brunsdon C, Radburn R (2011). A spatial analysis of variations in health access: linking geography, socio-economic status and access perceptions. Int J Health Geogr.

[26] Leibbrandt MWI.; de Villiers, L: Methodology: Report on NIDS Wave 1. 2009. Avaialbe at http://www.nids.uct.ac.za/publications/technical-papers/108-nids-technical-paper-no1/file. Accessed 23 Jan 2018.

[27] About NIDS. Available at http://www.nids.uct.ac.za/documentation/faqs/data-about-nids. Accessed 23 Jan 2018.

[28] SALDRU: Wave 4 data: southern Africa labour and development research unit National Income Dynamics Study 2014 - 2015, Wave 4 [dataset] Version 11 Cape Town: Southern Africa Labour and Development Research Unit [producer], 2017 Cape Town: DataFirst [distributor], 2017 2015.

[29] SALDRU: Wave 3 data: southern Africa labour and development research unit National Income Dynamics Study 2012, Wave 3 [dataset] Version 21 Cape Town: Southern Africa Labour and Development Research Unit [producer], 2017 Cape Town: DataFirst [distributor], 2017 2012.

[30] SALDRU: Wave 2 data: southern Africa labour and development research unit National Income Dynamics Study 2010-2011, Wave 2 [dataset] Version 31 Cape Town: Southern Africa Labour and Development Research Unit [producer], 2017 Cape Town: DataFirst [distributor], 2017 2011.

[31] SALDRU: Wave 1 data: southern Africa labour and development research unit National Income Dynamics Study 2008, Wave 1 [dataset] Version 61 Cape Town: Southern Africa Labour and Development Research Unit [producer], 2017 Cape Town: DataFirst [distributor], 2017 2008.

[32] About National Geo-spatial Information (NGI). [ONLINE] Available at: http://www.ngi.gov.za/. Accessed 4 Sept 2017.

[33] ESRI: Environmental Systems Research Institute (ESRI)**.** ArcGIS 10**.**0**.** Redlands**,** CA. 2010.

[34] Ferguson WJ, Kemp K, Kost G (2016). Using a geographic information system to enhance patient access to point-of-care diagnostics in a limited-resource setting. Int J Health Geogr.

[35] Delamater PL, Messina JP, Shortridge AM, Grady SC (2012). Measuring geographic access to health care: raster and network-based methods. Int J Health Geogr.

[36] Mokhele T, Weir-Smith G, Labadarios D. Development of health density indicators in South Africa using GIS. Conference paper by the Human Sciences Research Council (HSRC). 2012. Available from http://www.hsrc.ac.za/en/research-data/view/6105. Accessed 15 Jan 2018.

[37] Vasan RS, Larson MG, Leip EP, Evans JC, O'Donnell CJ, Kannel WB, Levy D (2001). Impact of high-normal blood pressure on the risk of cardiovascular disease. N Engl J Med.

[38] Anderson KM, Wilson P, Odell PM, Kannel WB (1991). An updated coronary risk profile. A statement for health professionals. Circulation.

[39] World Health Organization (WHO)**.** World Health Statistics 2012 Geneva: World Health Organization Available at **:** http://www.who.int/gho/publications/world_health_statistics/2012/en/. Accessed 13 Sept 2017.

[40] Mayosi B, Bryer A, Lambert V, Levitt N, Noakes T, Ntsekhe M, Opie L, Rayner B, Zilla P, Abrahams Z. A statement of intent on the formation of the NCRP on cardiovascular and metabolic disease: a new initiative to fight heart disease, stroke, diabetes and obesity in south. Africa. 2007; 17392988

[41] Chobanian AV, Bakris GL, Black HR, Cushman WC, Green LA, Izzo JL, Jones DW, Materson BJ, Oparil S, Wright JT (2003). Seventh report of the joint national committee on prevention, detection, evaluation, and treatment of high blood pressure. Hypertension.

[42] Maimaris W, Paty J, Perel P, Legido-Quigley H, Balabanova D, Nieuwlaat R, Mckee M (2013). The influence of health systems on hypertension awareness, treatment, and control: a systematic literature review. PLoS Med.

[43] Shi L, MacLeod KE, Zhang D, Wang F, Chao MS (2017). Travel distance to prenatal care and high blood pressure during pregnancy. Hypertens Pregnancy.

[44] Gielen S, Laughlin MH, O’Conner C, Duncker DJ (2015). Exercise training in patients with heart disease: review of beneficial effects and clinical recommendations. Prog Cardiovasc Dis.

[45] Chow CK, Jolly S, Rao-Melacini P, Fox KA, Anand SS, Yusuf S (2010). Association of diet, exercise, and smoking modification with risk of early cardiovascular events after acute coronary syndromes. Circulation.

[46] Halm EA, Lee C, Chassin MR (2002). Is volume related to outcome in health care? A systematic review and methodologic critique of the literature. Ann Intern Med.

[47] Nteta TP, Mokgatle-Nthabu M, Oguntibeju OO (2010). Utilization of the primary health care services in the Tshwane region of Gauteng Province, South Africa. PLoS One.

[48] Kapwata T, Morris N, Campbell A, Mthiyane T, Mpangase P, Nelson KN, Allana S, Brust JCM, Moodley P, Mlisana K (2017). Spatial distribution of extensively drug-resistant tuberculosis (XDR TB) patients in KwaZulu-Natal, South Africa. PLoS One.

[49] National and provincial labour market, Statistical release P0211.4.2. Available at https://www.statssa.gov.za/publications/P02114.2/P02114.22015.pdf. [Accesed on 28 Nov 2017].

[50] World Health Organization (WHO). Preventing chronic diseases:a vital investment. Available at : http://www.who.int/chp/chronic_disease_report/contents/en/. Accessed 12 Sept 2017.

[51] Sliwa K, Acquah L, Gersh BJ, Mocumbi AO (2016). Impact of socioeconomic status, ethnicity, and urbanization on risk factor profiles of cardiovascular disease in Africa. Circulation.

[52] Marshall IJ, Wang Y, Crichton S, McKevitt C, Rudd AG, Wolfe CD (2015). The effects of socioeconomic status on stroke risk and outcomes. Lancet Neurol.

[53] Barr DA (2017). The childhood roots of cardiovascular disease disparities. Mayo Clin Proc.

