# Supplementary figures and images for: Geographic assessment of access to health care in patients with cardiovascular disease in South Africa
Source: BMC Health Serv Res. 2018 Mar 22;18:197. doi: 10.1186/s12913-018-3006-0 (PMC5863828; doi:10.1186/s12913-018-3006-0)

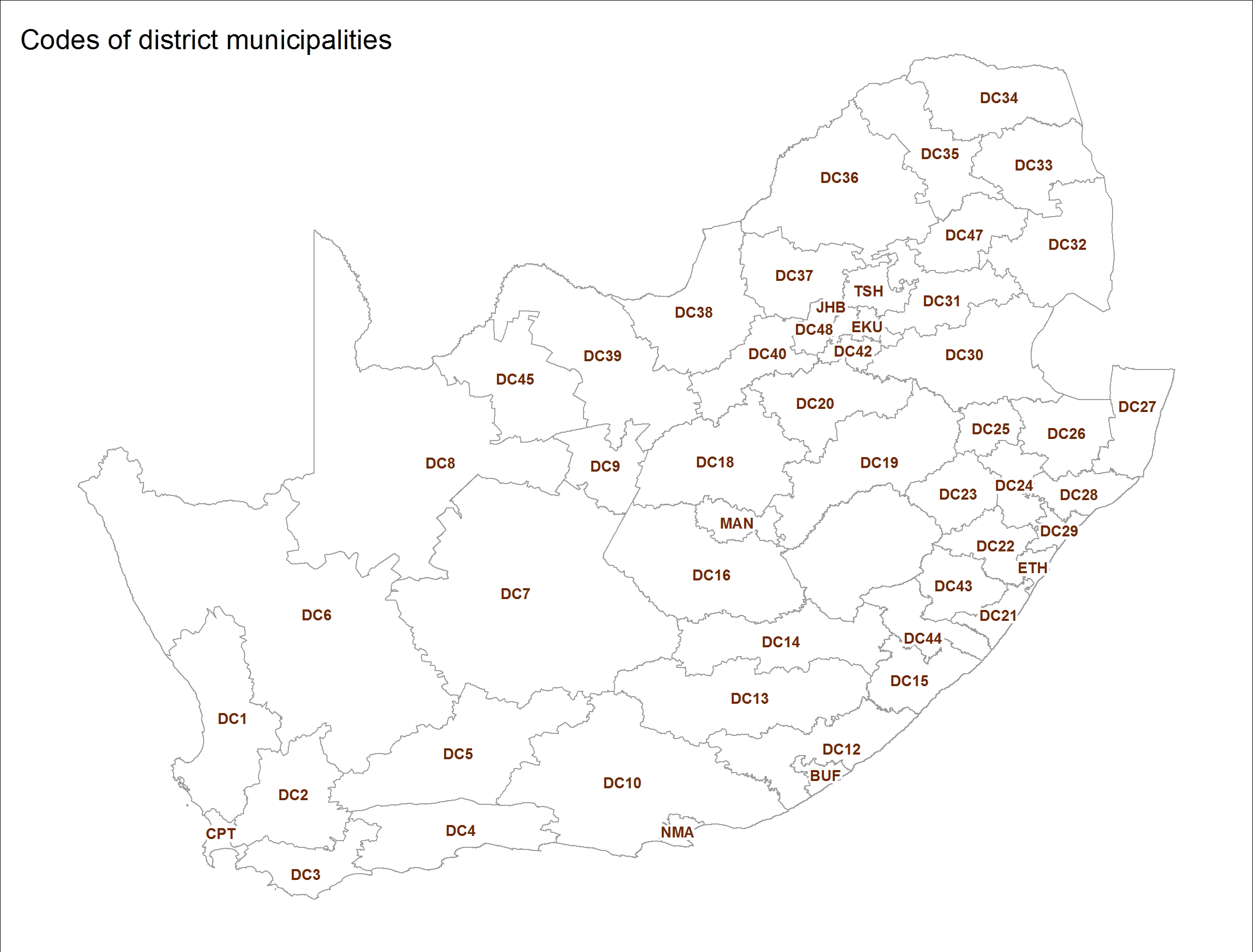

Supplement: Supplementary file 1 — Graphs showing mean distance to a health facility from a household with subject diagnosed with a heart condition for each of the waves by district. Part 1: Codes of district municipalities. Map of South Africa displaying district codes. Part 2: Mean distance in kilometres to a health facility from a household with subject diagnosed with a heart condition in the 2008 and 2010–2011 NIDS Survey wave by district, South Africa. Mean distance to health facility by district for each wave. Part 3: Mean distance in kilometres to a health facility from a household with subject diagnosed with a heart condition in the 2012 and 2014–2015 NIDS Survey wave by district, South Africa. Mean distance to health facility by district for each wave. (ZIP 707 kb) [file 12913_2018_3006_MOESM1_ESM.zip › Appendix 1 part 1R3.jpg]

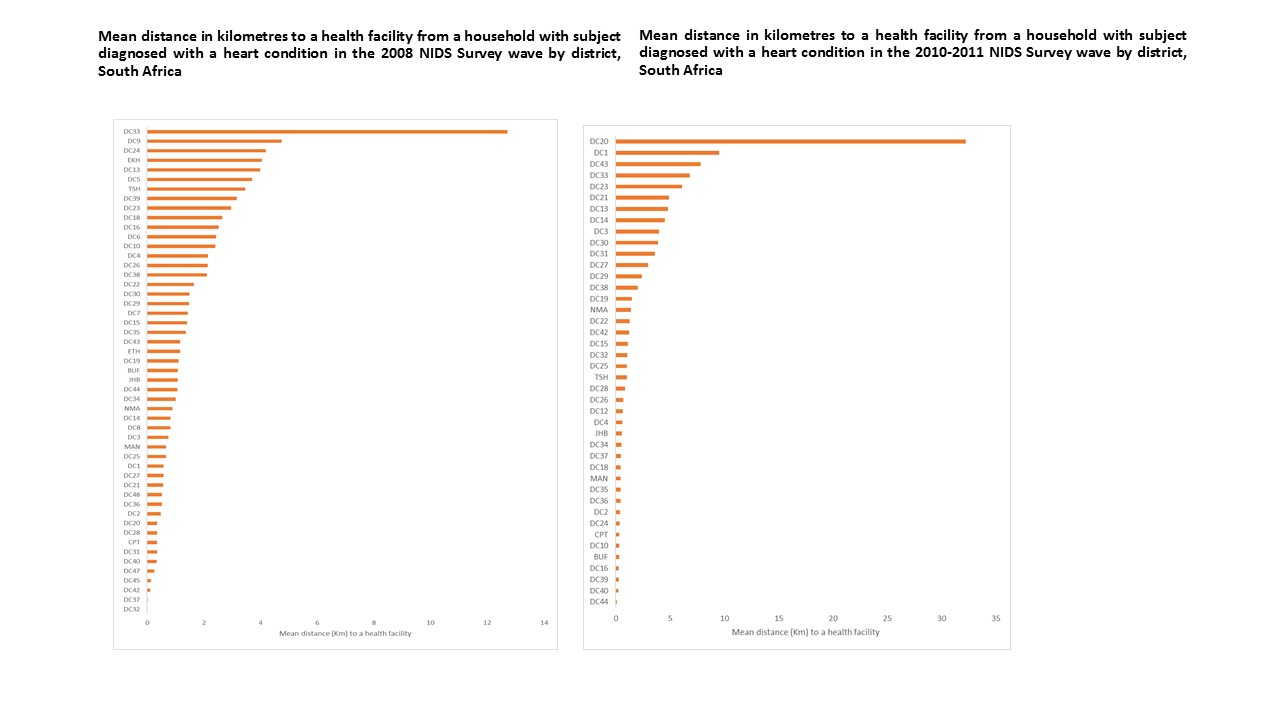

Supplement: Supplementary file 1 — Graphs showing mean distance to a health facility from a household with subject diagnosed with a heart condition for each of the waves by district. Part 1: Codes of district municipalities. Map of South Africa displaying district codes. Part 2: Mean distance in kilometres to a health facility from a household with subject diagnosed with a heart condition in the 2008 and 2010–2011 NIDS Survey wave by district, South Africa. Mean distance to health facility by district for each wave. Part 3: Mean distance in kilometres to a health facility from a household with subject diagnosed with a heart condition in the 2012 and 2014–2015 NIDS Survey wave by district, South Africa. Mean distance to health facility by district for each wave. (ZIP 707 kb) [file 12913_2018_3006_MOESM1_ESM.zip › Appendix 1 part 2R3.jpg]

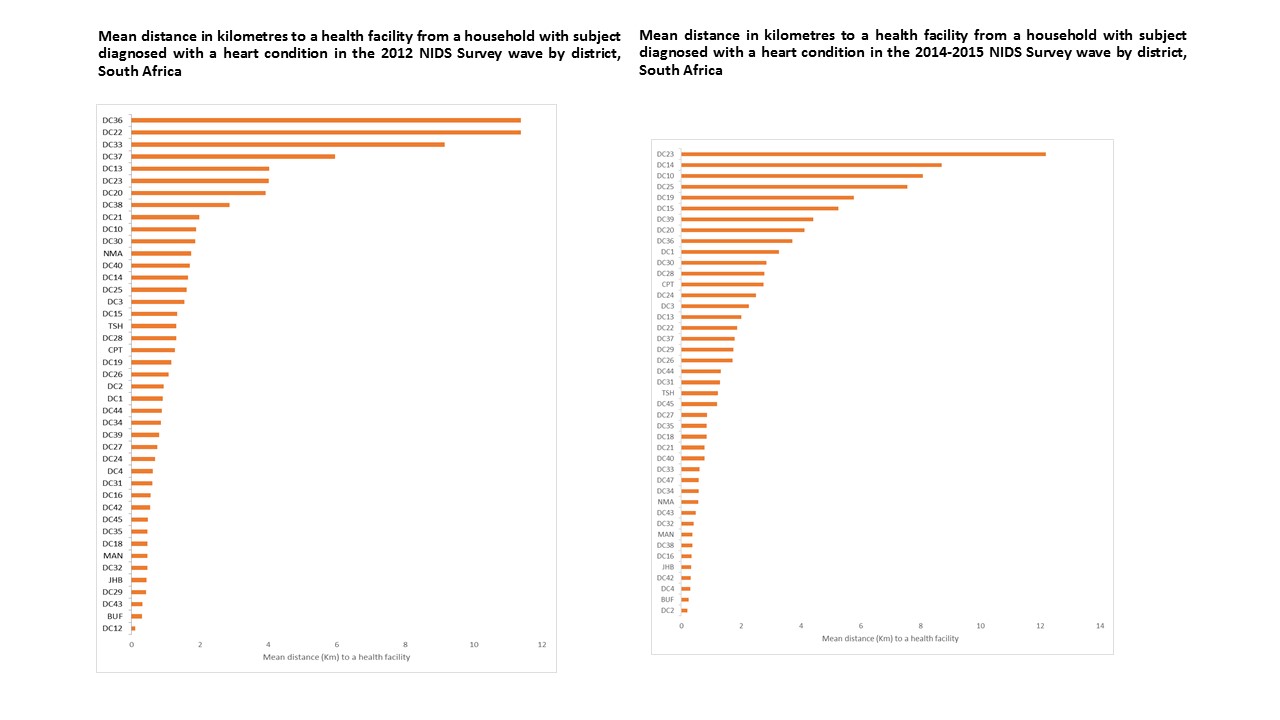

Supplement: Supplementary file 1 — Graphs showing mean distance to a health facility from a household with subject diagnosed with a heart condition for each of the waves by district. Part 1: Codes of district municipalities. Map of South Africa displaying district codes. Part 2: Mean distance in kilometres to a health facility from a household with subject diagnosed with a heart condition in the 2008 and 2010–2011 NIDS Survey wave by district, South Africa. Mean distance to health facility by district for each wave. Part 3: Mean distance in kilometres to a health facility from a household with subject diagnosed with a heart condition in the 2012 and 2014–2015 NIDS Survey wave by district, South Africa. Mean distance to health facility by district for each wave. (ZIP 707 kb) [file 12913_2018_3006_MOESM1_ESM.zip › Appendix 1 part 3R3.jpg]
